# Sacral neuromodulation improves bowel function in patients with low anterior resection syndrome

**DOI:** 10.1007/s10151-025-03263-8

**Published:** 2026-02-09

**Authors:** Amina Issa, Dean Lutrin, Shani Yarchi, Ruth Skvortsov, Rashid Isa, Yael Weksler, Lior Segev, Nir Horesh, Edward Ram, Ido Nachmany, Dan Carter

**Affiliations:** 1 Department of General Surgery, Sheba Medical Center, 2 Derech Sheba, 52621 Ramat Gan, Israel; 2 Pelvic Floor Disorders Unit, Sheba Medical Center, Ramat Gan, Israel; 3 Department of Colorectal Surgery, Cleveland Clinic Florida, Weston, FL USA; 4 Faculty of Medicine, Tel Aviv University, Tel Aviv, Israel

**Keywords:** Sacral neuromodulation, Low anterior resection syndrome (LARS), Fecal incontinence, Quality of life, Wexner score

## Abstract

**Background:**

Low anterior resection syndrome (LARS) is a common postoperative sequala affecting up to 70% of patients following rectal cancer surgery. Characterized by urgency, frequency, incontinence, and incomplete evacuation, LARS can significantly impair quality of life. Sacral neuromodulation (SNM) has emerged as a potential therapy for patients with refractory symptoms.

**Methods:**

This retrospective single-center study included 43 patients with LARS unresponsive to conservative therapy who underwent SNM between 2017 and 2025. All patients received the InterStim™ device following a positive test phase. Demographic, clinical, and surgical data were collected and analyzed. The primary outcomes were changes in LARS and Wexner incontinence scores. Secondary outcomes included changes in quality of life measured by the Fecal Incontinence Quality of Life (FIQL) questionnaire and Patient-Reported Outcomes Measurement Information System Global-10 (PROMIS-10) survey.

**Results:**

LARS scores improved from a mean of 37.9–29.9 (*p* < 0.001) and Wexner scores from 14.2 to 10.4 (*p* < 0.001). FIQL scores improved significantly across all subdomains: lifestyle (1.49–2.28, *p* < 0.001), coping (1.39–2.26, *p* < 0.001), depression (1.66–2.38 *p* < 0.001), and embarrassment (1.58–2.40, *p* < 0.001). PROMIS-10 scores also improved, with physical health increasing from 35.7 to 41.4 (*p* = 0.01) and mental health from 40.3 to 45.1 (*p* = 0.02). Six patients required device revision, and three experienced minor complications.

**Conclusions:**

SNM significantly improves bowel function and quality of life in patients with LARS refractory to conservative management and represents a promising therapeutic option.

## Introduction

Low anterior resection syndrome (LARS) is a frequent postoperative complication affecting up to 70% of patients following rectal cancer surgery [1, 2]. It encompasses a constellation of bowel dysfunction symptoms—fecal incontinence, urgency, frequency, and incomplete evacuation—that can persist long after surgery and profoundly impact quality of life [2, 3].

The pathophysiology of LARS is multifactorial, involving altered rectal compliance, impaired reservoir function and pelvic nerve damage, often exacerbated by neoadjuvant therapy and correlated with surgical factors including low anastomosis or prolonged period of bowel diversion [3]. Evaluation of LARS severity is typically based on validated scoring tools, including the LARS score and Wexner incontinence score [4, 5], alongside quality-of-life instruments such as the Fecal Incontinence Quality of Life (FIQL) questionnaire [6].

First-line treatments often include conservative measures such as dietary modifications and medications such as loperamide and ramosetron [2]. For patients with persistent symptoms, pelvic floor rehabilitation, biofeedback training, and transanal irrigation may offer additional benefit [2, 7]. However, for refractory cases, more invasive interventions, such as sacral neuromodulation (SNM) or even colostomy, may be required [2, 8].

Sacral neuromodulation is a minimally invasive technique that involves electrical stimulation of the sacral nerve roots to modulate pelvic floor function [9]. The procedure typically consists of two phases: a test phase where an electrode is implanted in a sacral foramen to evaluate clinical improvement, followed by permanent implantation of a neurostimulator if the test phase is successful [9]. Though its efficacy is well established in fecal incontinence, its role in LARS remains less defined [1, 10].

This study aims to evaluate the clinical outcomes of SNM in patients with LARS unresponsive to conservative measures. We specifically assess changes in bowel function, continence, and quality of life using validated symptoms and functional outcome measures.

## Methods

### Study population and design

This was a single center retrospective study of a prospectively maintained database of 44 patients with LARS who underwent SNM at the Sheba Medical Center between January 2017 and January 2025. One patient died of an unknown cause and was lost to follow-up. LARS was diagnosed using the LARS score, with a threshold greater than 20, with scores of 21–29 classified as minor LARS and scores of 30–42 classified as major LARS [4]. All patients had persistent LARS symptoms despite optimal conservative management and were evaluated by a multidisciplinary pelvic floor board for SNM candidacy. The study was approved by the institutional review board with registration number 8386-21SMC. The primary outcomes were changes in LARS and Wexner scores from baseline to post treatment. Secondary outcomes included changes in FIQL subdomains, PROMIS-10 physical and mental health scores, patient satisfaction, and complication or revision rates.

### Preoperative evaluation

Eligible patients were those with persistent symptoms despite prior treatments, including pelvic floor physiotherapy, biofeedback, and medications. Before SNM implantation, all patients underwent a thorough preoperative assessment, including anorectal manometry, transrectal ultrasound, and colonoscopy to characterize anorectal function and exclude structural abnormalities. Symptom severity was quantified using the LARS score and the Cleveland Clinic Incontinence Score (Wexner score), whereas quality of life was assessed with the FIQL questionnaire and the PROMIS-10 global health survey, which is validated in the Hebrew-speaking population [4, 5, 11]. Patient satisfaction and adverse events were systematically assessed through structured telephone interviews and clinic visits both before and after SNM therapy.

### Surgical procedure

The SNM procedure was performed in two distinct phases. The initial test phase was conducted under general anesthesia, during which a quadripolar tined lead was implanted in the S3 foramen, guided by continuous fluoroscopy and confirmed by observing appropriate motor responses such as anal contraction and hallux flexion. The lead was connected to an external stimulator, and patients were monitored weekly in the clinic for symptom assessment and wound care. Clinical assessment was conducted by directly questioning patients regarding their bowel habits before and after the temporary stimulation phase. Patients who achieved a clinically meaningful improvement—defined as a reduction of more than 50% in the average number of daily bowel movements—were considered eligible for permanent implantation, which was performed 3 weeks after completion of the temporary phase. The permanent phase was performed under local anesthesia, with the neurostimulator placed in a subcutaneous gluteal pocket and programmed according to patient feedback and clinical response.

### Statistical analysis

All data were collected from our department’s database, medical records, and telephone interviews and included in a specific database, respecting all data protection regulations. Statistical analysis was performed by a biostatistician using Python 3.13.3 software. A descriptive analysis was performed for patient characteristics. For analytic comparisons (pre-/post-SNM variables), correlations were performed for continuous variables and difference of means for qualitative variables. Stuart–Maxwell chi-square tests, Spearman’s rho (correlation) and Mann–Whitney *U* or Wilcoxon *W* tests for mean comparisons were performed when appropriate. To analyze changes in LARS and Wexner scores related to specific variables, nonparametric correlations were performed (rho, *W*, and *U*). All tests were two-tailed, with statistical significance set at *p* < 0.05.

## Results

A total of 43 patients with LARS who underwent sacral neuromodulation were included. The median age was 67 years (range 44–87 years), and 69.8% were male. Common comorbidities included diabetes mellitus (62.9%), hyperlipidemia (48.6%), hypertension (37.1%), and ischemic heart disease (14.3%).

The indication for low anterior resection was malignant disease in 93.0% of patients. Total mesorectal excision (TME) was performed in 82.9%, whereas 17.1% underwent partial mesorectal excision. End-to-end anastomosis was the most common type (88.9%), and a diverting ileostomy was created in 90.7%. Anastomotic leakage occurred in 8.3%, and 7.9% of patients required surgical reintervention. Total neoadjuvant therapy was administered to 12 patients (27.9%) and standard chemoradiotherapy to 25 patients (58.1%).

The mean tumor level from the anal margin was 7.81 cm (range 1–20 cm, standard deviation of 3.74). Two patients had minor LARS (scores 21–29), while 41 patients had major LARS (scores 30–42). Previous rectal surgeries were reported in four patients (9.3%), all categorized as “other” (no cases of hemorrhoidectomy, anal fistula, or anal fissure). Demographic, clinical, and preoperative details are reported in Table 1.

**Table 1:** Patients’ demographics and disease characteristics

Variable	*n* = 43
Median age in years, (range)	67 (44–87)
Gender	
Male *n* (%)	30 (69.8%)
Female *n* (%)	13 (30.2%)
BMI mean (range)	25.5 (17.8–34.4)
Comorbidities prevalence	
DM, *n* (%)	22 (62.9%)
HTN, *n* (%)	13 (37.1%)
HLP, *n* (%)	17 (48.6%)
IHD, *n* (%)	5 (14.3%)
Asthma, *n* (%)	2 (5.7%)
Heart failure, *n* (%)	1 (2.9%)
CKD, *n* (%)	1 (2.9%)
Other, *n* (%)	17 (48.6%)
Blood thinners, *n* (%)	9 (23.1%)
LAR surgery details	
Malignant indication (LAR)	40 (93.0%)
TME	29 (82.9%)
End-to-end anastomosis	32 (88.9%)
Protective ileostomy	39 (90.7%)
Total neoadjuvant therapy	12 (27.9%)
Standard chemoradiotherapy	25 (58.1%)

BMI, body mass index; CKD, chronic kidney disease; DM, diabetes mellitus; HLP, hyperlipidemia; HTN, hypertension; IHD, ischemic heart disease; LAR surgery details, low anterior resection surgery details; LAR, malignant indication for low anterior resection (malignant indication); TME, total mesorectal excision

One patient failed the initial test phase and did not get to the second phase. Postoperative complications occurred in three patients (7%): one superficial wound infection treated with antibiotics and two cases of lead extrusion (one resolved with revision, one required device removal). Device revision was required in six patients (14%) due to lead displacement (*n* = 2), battery depletion (*n* = 2), device upgrade (*n* = 1), or unknown reasons (*n* = 1). The median follow-up was 22.9 (3–91.3) months.

### SNM implantation outcomes

**Fig. 1:**
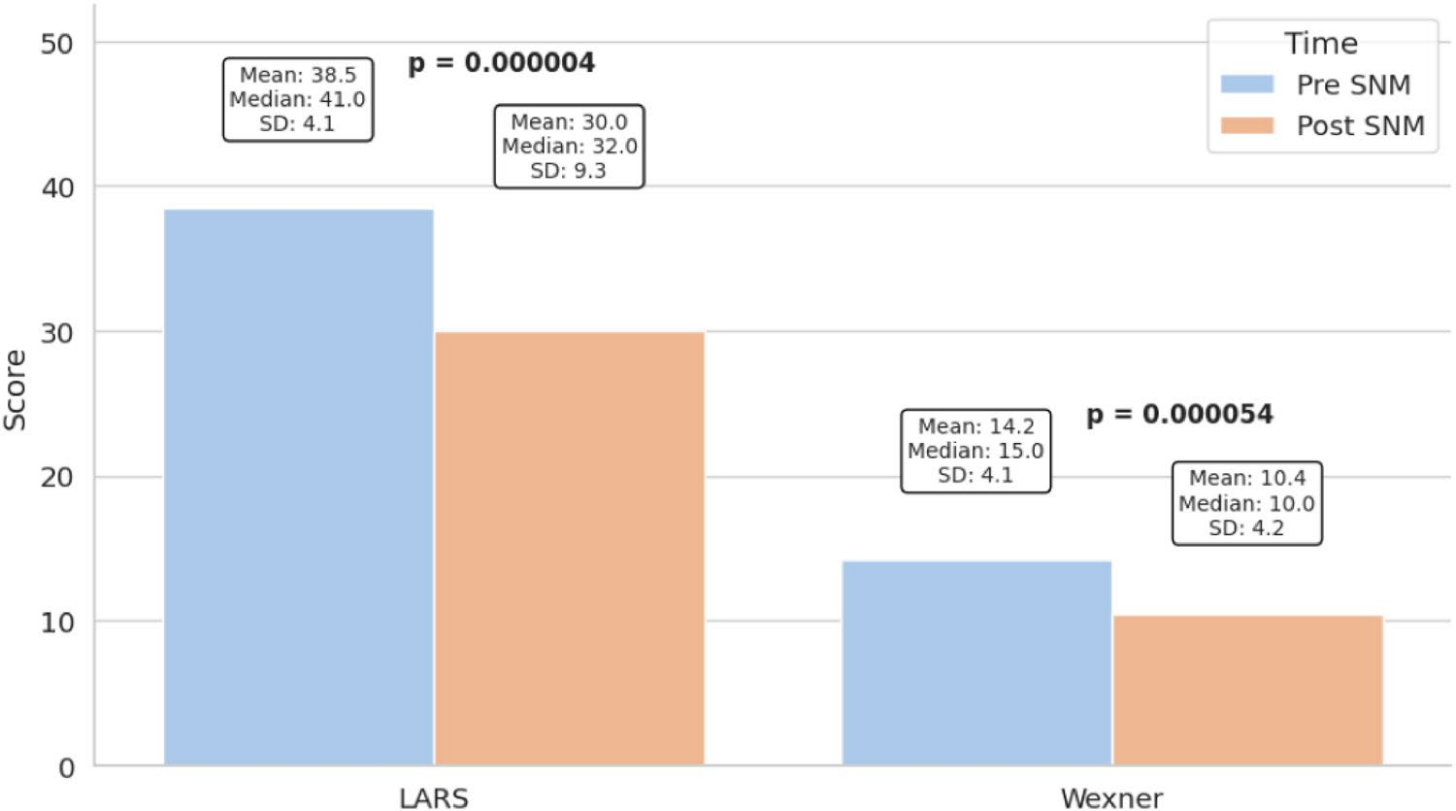
Effect of SNM on LARS and Wexner scores

Sacral neuromodulation resulted in significant improvements in both bowel function and quality of life. The mean LARS score decreased from 37.9 ± 9.3 to 29.9 ± 6.8 (*p* < 0.001), and 57.1% of patients reported a marked improvement in bowel frequency and urgency, whereas 3.6% experienced worsening of urgency (*p* < 0.001), and none experienced worsening of frequency (*p* = 0.0017). The remaining patients reported no change. The Wexner incontinence score improved from 14.2 ± 10.4 to 10.4 ± 6.8 (*p* < 0.001) (Fig. 1).

**Fig. 2:**
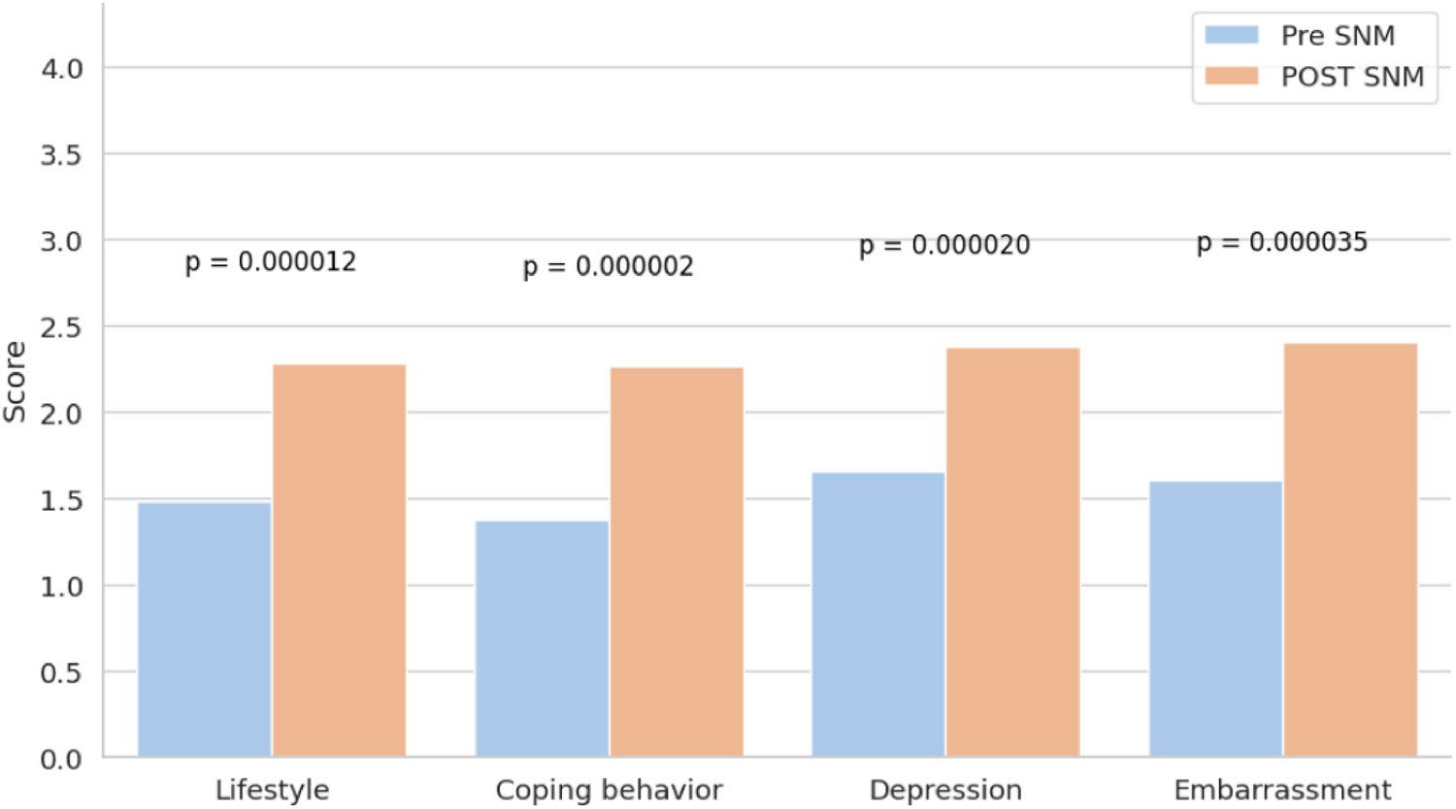
Fecal incontinence quality of life

FIQL questionnaire demonstrated statistically significant improvements across all subdomains. The lifestyle score increased from 1.49 ± 0.58 to 2.28 ± 0.90, the coping domain from 1.39 ± 0.44 to 2.26 ± 0.92, the depression domain from 1.66 ± 0.63 to 2.38 ± 0.88, and the embarrassment score from 1.58 ± 0.70 to 2.40 ± 0.90 (all *p* < 0.001) (Fig. 2).

**Fig. 3:**
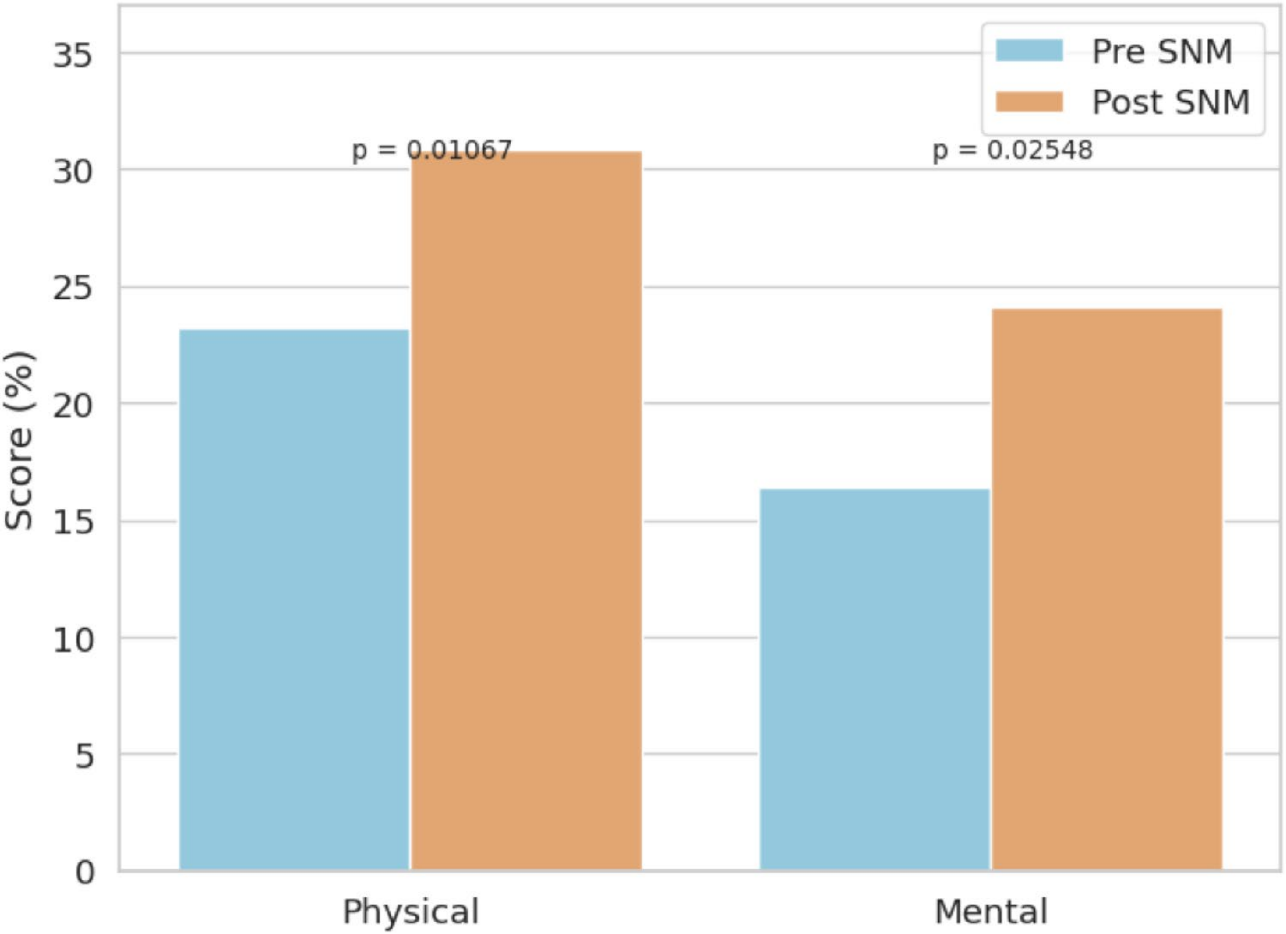
Global physical and mental scores

Similarly, PROMIS-10 global health scores showed significant gains. The physical health score improved from 35.7 ± 5.9 to 41.4 ± 6.6 (*p* = 0.010), whereas the mental health score increased from 40.3 ± 7.4 to 45.1 ± 7.9 (*p* = 0.025) (Fig. 3).

## Discussion

Sacral neuromodulation is emerging as a promising intervention for patients suffering from LARS, a debilitating condition that can severely compromise quality of life following rectal cancer surgery. In this study, SNM was associated with significant improvements in bowel function, continence, and patient-reported quality-of-life measures among individuals with LARS refractory to conservative therapy. The magnitude of symptom improvement observed in our cohort reflects meaningful clinical benefit, consistent with prior studies evaluating SNM for fecal incontinence and LARS [1, 12–17]. Both the LARS and Wexner scores showed statistically significant reductions, reflecting tangible gains in continence, urgency, and stool frequency. Importantly, these improvements translated into better daily functioning and emotional health, as evidenced by significant gains across all subscales of the FIQL questionnaire—including lifestyle, coping, depression, and embarrassment domains. While the PROMIS-10 is a validated and psychometrically robust global health measure, particularly within gastrointestinal populations, it has not yet been established in the literature as a standard or primary outcome in studies assessing sacral neuromodulation for fecal incontinence [11]. Nevertheless, in our study, PROMIS-10 results highlighted the broader impact of SNM, with patients reporting meaningful improvements in both physical and mental health following treatment—underscoring the therapy’s holistic benefit beyond symptom control.

Our findings align with previous reports suggesting SNM modulates afferent neural pathways and restores pelvic floor reflexes impaired by rectal resection and radiotherapy [5, 18, 19]. The consistently low complication rate in our series further supports the safety of SNM, with only minor adverse events such as superficial infection or lead extrusion, all of which were managed successfully [20–23].

These findings are further reinforced by the recent SANLARS randomized controlled trial, the most robust clinical evidence to date evaluating SNM in patients with LARS [12]. In this multicenter, double-blind, crossover study, Marinello et al. demonstrated that SNM led to significant improvements in LARS and continence scores, as well as reductions in urgency, clustering, and bowel emptying difficulties, with sustained benefits at 6- and 12-month follow-up. Importantly, their trial also emphasized improvements in quality of life, mirroring the multidimensional gains observed in our cohort. The SANLARS study provides critical prospective validation of SNM’s therapeutic role in LARS and supports its consideration earlier in the treatment algorithm for patients with refractory symptoms. Nevertheless, the trial also highlighted important limitations, as the expected 40% reduction in LARS scores at 12 months was not achieved, raising concerns about long-term durability [24]. Moreover, reliance on the LARS score as the sole outcome measure may not fully capture functional outcomes, with continence scores and bowel diaries offering a more comprehensive assessment. Reduced effectiveness in patients with pelvic fibrosis or anastomotic complications further underscores the need for subgroup analyses and prospective validation, whereas the absence of extended follow-up beyond 12 months limits conclusions regarding sustained efficacy and cost-effectiveness.

This study contributes to the growing body of evidence supporting the integration of SNM into the treatment algorithm for LARS. Although SNM has traditionally been reserved for treating fecal incontinence, our findings suggest that its role should be expanded to include carefully selected patients with LARS, particularly those who do not respond to dietary modifications, pharmacologic interventions, or pelvic floor therapies. However, several limitations should be acknowledged. This was a retrospective, single-center study, which may limit the generalizability of the results. The absence of a control group restricts the ability to draw causal inferences about SNM’s efficacy, and the relatively short follow-up period prevents evaluation of long-term outcomes and the durability of treatment benefits. Given these considerations, long-term prospective, multicenter trials are warranted to confirm the durability of SNM, define its cost-effectiveness, and establish its optimal place within the therapeutic algorithm for LARS.

In conclusion, SNM significantly improves bowel function and quality of life in patients with LARS who have failed conservative treatment. It offers a safe, minimally invasive, and effective therapeutic option that addresses both the physiological and psychosocial burden of this condition.

## Data Availability

No datasets were generated or analyzed during the current study.
